# Removal of Cadmium (II) from Aqueous Solution Using *Galdieria sulphuraria* CCMEE 5587.1

**DOI:** 10.3390/biotech13030028

**Published:** 2024-08-01

**Authors:** Hari Lal Kharel, Lina Jha, Melissa Tan, Thinesh Selvaratnam

**Affiliations:** 1 Department of Civil and Environmental Engineering, Lamar University, Beaumont, TX 77705, USA; hkharel@lamar.edu (H.L.K.); ljha@lamar.edu (L.J.); mtan2@lamar.edu (M.T.); 2 Center for Advances in Water & Air Quality, College of Engineering, Lamar University, Beaumont, TX 77705, USA

**Keywords:** *Galdieria sulphuraria*, cadmium removal, bioremediation, acidic pH, microalgae

## Abstract

The release of cadmium into the environment is a significant global concern due to its toxicity, non-biodegradability, and persistence in nature. There is an urgent need for effective, eco-friendly, and cost-effective systems for removing Cd because of the many drawbacks of conventional physicochemical techniques. This study investigated the ability of the extremophile red microalgal strain *Galdieria sulphuraria* CCMEE 5587.1 to tolerate and remove Cd (II) ions at acidic pH in a controlled laboratory environment. Three distinct concentrations of Cd (1.5 mg L^−1^, 3 mg L^−1^, and 6 mg L^−1^) were introduced to the cyanidium medium, and *G. sulphuraria* cells were introduced in the medium and grown for ten days. Four distinct aspects were identified regarding Cd removal: time course Cd removal, total Cd removal, extracellular Cd removal, and intracellular Cd removal. The inhibitory effects of Cd on *G. sulphuraria* growth were observed using a daily growth profile. Initial incubation days showed an inhibition of *G. sulphuraria* growth. In addition, increasing the Cd concentration in the medium decreased the growth rate of *G. sulphuraria*. Rapid Cd removal occurred on the first day of the experiment, followed by a steady removal of Cd until the last day. The highest total removal efficiency occurred in a medium containing 3 mg L^−1^ of Cd ions, which was 30%. In contrast, the highest sorption capacity occurred in a medium containing 6 mg L^−1^ of Cd ions, which was 1.59 mg g^−1^ of dry biomass. In all media compositions, a major fraction (>80%) of Cd removal occurred via adsorption on the cell surface (extracellular). These results showed that *G. sulphuraria* cells can remove Cd ions from aqueous solution, which makes them a potential bioremediation option for heavy metal removal.

## 1. Introduction

The rapid increase of industrialization and urbanization has led to significant wastewater discharge into natural water ecosystems. This wastewater is a byproduct of various industrial processes that heavily utilize metals, resulting in a substantial release of metal-containing waste into the environment. Industries such as metal mining, oil and textile, metallurgy, electroplating, galvanization, and metal surface treatment are among the major contributors to the release of heavy metals (HMs) into the environment [1,2]. 

Cadmium (Cd) is a very poisonous, persistent, and toxic inorganic HM inherent to the Earth’s crust. Cd is recognized as one of the most hazardous environmental pollutants and non-threshold toxins [3]. The concentration of Cd in the environment is steadily rising as a result of its extensive use in industrial processes [4]. Cd is released into the environment from several industrial applications, such as in making phosphors for TV tubes and batteries, electroplating, preparing alloys, melting, dyeing, mining, and refining petrochemicals. Consequently, this has increased Cd levels in aquatic ecosystems [4,5]. According to the US Environmental Protection Agency (EPA), the maximum allowable concentration of Cd in drinking water is 0.005 mg L^−1^ [6,7]. The regulation limit of Cd in wastewater effluent is variable and depends on various factors, including the industry’s type and purpose, control technology, and disposal technique. For example, according to the US EPA—in 40 CFR part 437—the maximum allowable limits of Cd in centralized wastewater treatment effluent cannot exceed 0.163 mg L^−1^ [8]. 

Cd exists naturally in ultra-trace quantities, but its severe toxicity and bioaccumulation in living organisms create significant concern. It poses a significant risk to human beings and aquatic creatures due to its acute toxicity [5]. Toxic effects of Cd bioaccumulation on human health include nausea, vomiting, diarrhea, muscle cramps; and increased risks to the lungs, and the cardiovascular and nervous systems [9,10]. Therefore, Cd removal from wastewater prior to environmental release is essential. 

Various techniques—including adsorption, precipitation, electro-coagulation, advanced oxidation, nano/ultrafiltration, reverse osmosis, and electrochemical technologies—have been employed to remove HMs from contaminated aquatic environments [11,12,13]. However, these methods often prove inefficient at low metal concentrations, are ineffective, harmful to the environment, and can lead to secondary contamination [14]. Moreover, these techniques have a high initial investment, operating cost, and energy input requirement [15]. As a result, there is a need for more practical and environmentally friendly techniques for the removal of HMs from waste streams.

In recent years, phycoremediation has gained attention as a potential alternative to conventional physiochemical methods, as it is more cost-efficient, environmentally friendly, and sustainable. Phycoremediation techniques involve using microalgae to purify water and wastewater by exploiting their biological properties [16]. Algal biomass can bio-absorb nutrients, organics, and HMs; making it easier to dispose of waste or bio-transforming them into a non-hazardous form [17]. Microalgae have remarkable biological properties that have led to their greater usage in HM removal, such as requiring low nutrition, having a large surface area compared to volume, many binding sites, and high photosynthetic efficiency [18]. 

*Galdieria sulphuraria* (*G. sulphuraria*) is a unicellular, thermophilic, and acidophilic red alga that can survive in extreme conditions, such as high temperatures (37–55 °C) and extremely low pH (0.0–3.0) [19,20,21]. In addition, there is evidence that it can withstand HMs, which are prevalent in sulfur springs [22]. *G. sulphuraria* is best suited for bioaccumulation and bio-removal of HMs from aquatic environments, because it has the highest resistance to toxic HMs and rare earth elements (REEs) among eukaryotic algae [23,24]. Furthermore, *G. sulphuraria* demonstrates metabolic flexibility by effectively thriving on over 50 distinct carbon sources [25]. Due to the association of *G. sulphuraria* with such demanding biological features, it is being studied for its resistance to HMs and its potential in phycoremediation techniques [26]. In the past, *G. sulphuraria* has successfully been used to remove carbon and nutrients (NH_4_-N and PO_4_-P) from produced water, landfill leachate, and municipal wastewater [27,28,29].

Municipal wastewater and landfill leachate are typically rich in nutrients and contain trace levels of Cd [30]. Most of the HM removal techniques—such as ion exchange or lime precipitation—are inefficient and costly for low concentrations below 100 mg L^−1^ [2,14]. Furthermore, municipal wastewater treatment systems are susceptible to receiving highly acidic wastewater, including HMs [31,32]. The majority of algal strains thrive in neutral pH conditions, necessitating the dilution of acidic wastewater before bioremediation. This study aims to evaluate the efficiency of *G. sulphuraria* CCMEE 5587.1 in removing low concentrations of Cd (II) (0 mg L^−1^ to 6 mg L^−1^) at acidic pH. In addition, this study aims to measure the inhibitory effect of Cd ions on *G. sulphuraria* growth and biomass production. 

## 2. Materials and Methods

### 2.1. Algal Strain and Cultural Medium

This *G. sulphuraria* CCMEE 5587.1 strain was obtained from the Culture Collection of Microorganisms from Extreme Environments at the University of Oregon. The strain was cultured in an incubator (Percival, IA, USA) set at 40 °C with continuous lighting at 4000 lux for 24 h [33]. The growing process began with carefully streaking axenic cultures onto agar plates. Subsequently, individual colonies were selected for transfer to progressively larger flasks until they reached 4-L Erlenmeyer flasks [29]. The Cynadium medium (CM) was used as a growth medium, and its preparation involved the addition of the following macro- and micro-level ingredients: (NH_4_)_2_SO_4_: 1.32 g L^−1^; KH_2_PO_4_: 0.27 g L^−1^; NaCl: 0.12 g L^−1^; MgSO_4_·7H_2_O: 0.25 g L^−1^; CaCl_2_·2H_2_O: 0.07 g L^−1^; Nitch’s Trace Element Solution: 0.5 mL L^−1^; FeCl_3_ (solution = 0.29 g L^−1^): 1.0 mL L^−1^ (2023_Kharel). The pH of the medium was kept at 2.5 by adding 10 N H_2_SO_4_.

### 2.2. Preparation of Different Media Composition

Analytical grade Cd (NO_3_)_2_·4H_2_O salt was dissolved in distilled water to produce Cd (II) stock solution of 1000 mg L^−1^. The stock solution was then added to the CM in specific concentrations to achieve the three desired Cd concentrations (1.5 mg L^−1^, 3 mg L^−1^, and 6 mg L^−1^) and nutrients in the medium. 

### 2.3. Experimental Setup

Primarily, *G. sulphuraria* was harvested during the exponential growth phase. Then, it was centrifuged at 3000 rpm for 10 min at 4 °C using a Centrifuge 5920R. (Eppendorf AG 22331 Hamberg, Germany). The biomass was resuspended in various media compositions after centrifugation, while the supernatant was discarded. 

The experiment included three different Cd concentrations based on the probable concentration in real wastewater effluent. Two controls were developed for each concentration to exclude the possibility that the observed effects were due to factors other than *G. sulphuraria*. A positive control had *G. sulphuraria* and no Cd ions in the CM, while a negative control had Cd with no *G. sulphuraria*. All the tests were carried out in triplicate for ten days in 125 mL Erlenmeyer flasks with 50 mL of the experimental sample. All flasks were placed on a New Brunswick Innova 2050 platform shaker (Eppendorf, Edison, NJ, USA) within the incubator, and shaken at 120 rpm. The CO_2_ content in the incubator was kept at 3% *v*/*v*.

### 2.4. Measurement of Biomass Density

The biomass density was measured daily and evaluated by measuring the optical density (OD) at 750 nm using the HACH DR 3900 spectrophotometer (HACH, Loveland, CO, USA). The biomass density was measured in grams of ash-free dry weight per liter (g AFDW L^−1^) and related to OD at 750 nm using the following formula:

(1) $$Y=0.4775*X-0.0163,\;R^{2}=0.9967,\;n=12,\;r^{2}=0.997$$

Y = Ash-free dry wt.
X = OD value at 750 nm

### 2.5. Measurement of Time Course, Extracellular and Intracellular Cd Concentration

The samples were tested daily to determine the quantity of time course Cd removal. The algal cultures were centrifuged, and the 1 mL supernatant solutions were used to measure dissolved Cd concentrations in the supernatant. The determination of total, extracellular, and intracellular Cd removal was conducted on the final day of the experiment. After ten days of incubation, samples were collected and centrifuged at 6000 rpm with an AccuSpin 400 centrifuge (Fisher Scientific, D-77520, Osterode, Germany) for 10 min to separate the supernatant fraction and the cell fraction. The supernatant was filtered through a 0.45 µm-pore syringe filter and kept in a refrigerator for metal measurements while the cell fraction was used for further analysis. Inductively coupled plasma atomic emission spectroscopy (ICP-AES) (SHIMADZU ICPE 9820 simultaneous ICP atomic emission spectroscopy, Shimadzu, Kyoto, Japan) was used to determine the Cd concentration in each sample. The following equation was used to calculate the total removal efficiency of Cd ions from the solutions:

(2) $$\mathrm{Removal}\;\left(\%\right)=\frac{\left(C_{i}-C_{f}\right)}{C_{i}}$$

C_i_ = Cd concentrations in the supernatant on Day 0
C_f_ = Cd concentrations in the supernatant on Day 10

To isolate extracellular Cd removal, EDTA is used to remove Cd adsorbed onto the surface of the algal cells. The concentrated cell fraction was resuspended in 50 mL of 0.02 M EDTA and agitated for 10 min. The sample was again centrifuged, and the supernatant was filtered and analyzed to find the extracellular Cd removal. The concentration of intracellular Cd removal was calculated by using the following equation:

(3) $$R_{i}=R_{t}-R_{e}$$

R_i_ = Intracellular Cd removal
R_t_ = Total Cd removal
R_e_ = Extracellular Cd removal

### 2.6. Statistical Techniques and Graph Plotting

The experiment results were calculated from three separate biological replications and given as the mean of the repetitions ± standard deviation. The sample mean and standard deviation were found using Microsoft Excel. All of the featured graphs were plotted using ORIGINPRO 2024. Statistical differences were evaluated via a one-way ANOVA; a value of *p* ≤ 0.05 was considered significant.

## 3. Results

### 3.1. Cadmium Bioremediation

#### 3.1.1. Total Cadmium Removal

**Figure 1:**
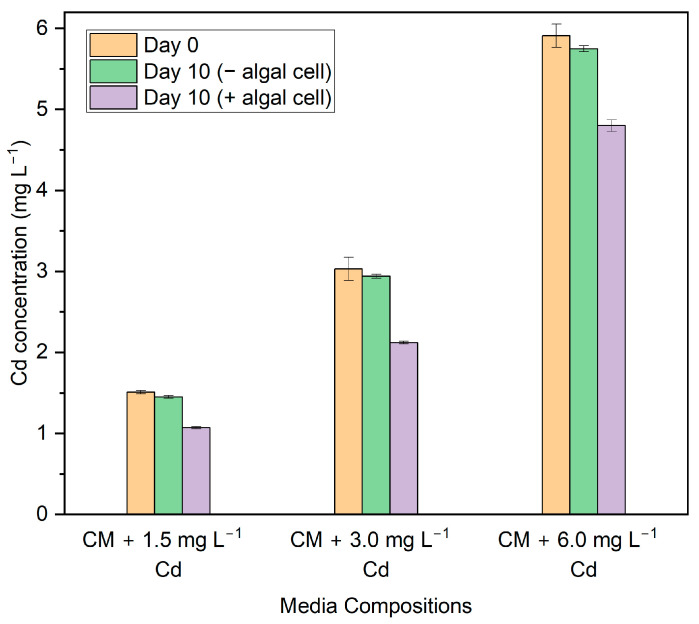
Cd concentration before and after the treatment in the medium. Data points represent the average ± SD of *n* = 3 biological replicates.

After ten days of being treated with living cells, *G. sulphuraria* was able to remove some extent of Cd from the medium. Figure 1 shows the results of the Cd concentration present in the medium before and after treatment during the experimental cycle. There was a significant amount of Cd reduction (*p* ≤ 0.05) in all media compositions on day 10. At all tested Cd concentrations, the total amount of Cd removed by *G. sulphuraria* increased directly to the Cd concentration. However, the removal efficiency of Cd decreased with increasing Cd concentration in the medium. There was no significant difference in removal efficiency (*p* > 0.05) (F = 0.042, *p* = 0.847) between 1.5 mg L^−1^ and 3 mg L^−1^. However, there was a significant difference in removal efficiency (*p* ≤ 0.05) (F = 19.977, *p* = 0.011) between 3 mg L^−1^ and 6 mg L^−1^. In a medium containing 3 mg L^−1^ Cd, *G. sulphuraria* achieves its maximum Cd removal effectiveness of 30.00%. Additionally, in the medium added with 1.5 mg L^−1^ and 6 mg L^−1^ of Cd, the removal effectiveness was 29.52% and 18.88%, respectively. The sorption capacity is maximum in a medium containing 6 mg L^−1^ Cd, which is 1.59 mg g^−1^ dry biomass. Similarly, the sorption capacity of *G. sulphuraria* in a medium containing 3 mg L^−1^ Cd and 1.5 mg L^−1^ Cd, is 1.3 and 0.63 mg g^−1^ dry biomass, respectively. 

#### 3.1.2. Time Course Cadmium Removal

**Figure 2:**
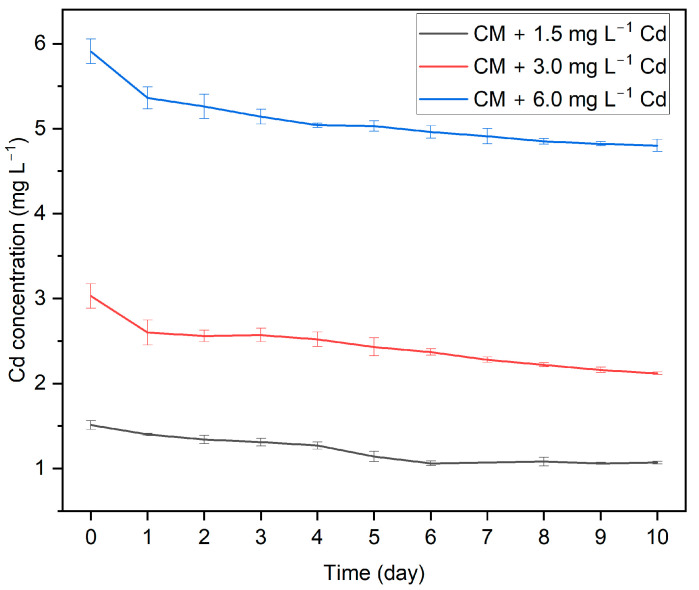
Cd concentration after the treatment in the medium with respect to exposure time. Data points represent the average ± SD of *n* = 3 biological replicates.

**Figure 3:**
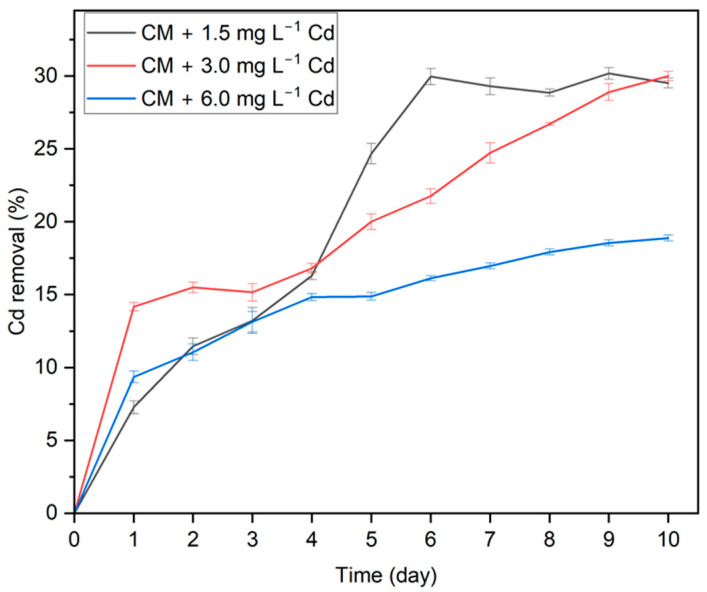
Cd removal percentage with respect to the exposure time. Data points represent the average ± SD of *n* = 3 biological replicates.

Figure 2 shows the total quantity of Cd that remained in the medium, and Figure 3 represents the Cd removal percentage when treated with *G. sulphuraria* at various Cd concentrations as a function of exposure time. The efficacy of Cd removal by *G. sulphuraria* increased rapidly on the first day and gradually increased during the entire experiment. After some days, the Cd removal rate decreased due to Cd ions binding to functional groups on cell surfaces, and the subsequent competition among Cd ions as the quantity of available binding sites decreased. In the medium added with 1.5 mg L^−1^ Cd, the concentration in the medium decreased until the sixth day, and then it was almost constant. However, in the medium added with 3 and 6 mg L^−1^ Cd, the concentration decreased until the last day of the experiment.

#### 3.1.3. Extracellular and Intracellular Cadmium Removal

**Figure 4:**
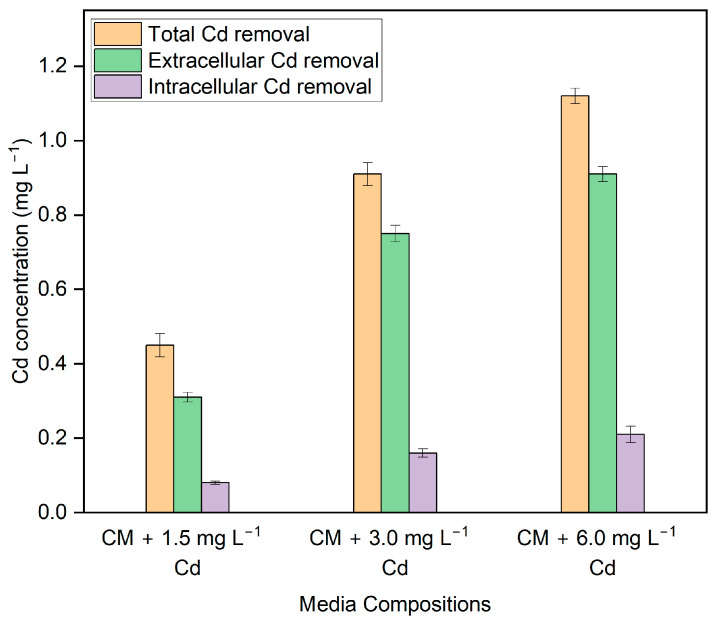
Fraction of Cd removal concentration. Data points represent the average ± SD of *n* = 3 biological replicates.

After coming into contact with HMs, the microalgal cells either adsorbed some of the metals onto their surface or allowed them to accumulate inside their own cells. Figure 4 shows the results of the fraction of extracellular and intracellular Cd removal in different media compositions. In all cultures, the quantity of Cd adsorbed on the surface of the cell was significantly higher (*p* ≤ 0.05) (F = 125.38, *p* = 0.0003 (1.5 mg L^−1^)), (F = 82.32, *p* = 0.0008 (3 mg L^−1^)), (F = 101.55, *p* = 0.0009 (6 mg L^−1^)) than the quantity removed intracellularly. The corresponding extracellular removal was 82.31%, 82.27%, and 81.31% in the medium having Cd concentration of 1.5 mg L^−1^, 3 mg L^−1^, and 6 mg L^−1^, respectively. This study found that after 10 days of being exposed to Cd, the amount of Cd adsorbed on the surface of *G. sulphuraria* was significantly larger than the amount that accumulated inside the cells. The results show that Cd removal by *G. sulphuraria* is largely due to a non-metabolic process.

### 3.2. Growth of G. sulphuraria

**Figure 5:**
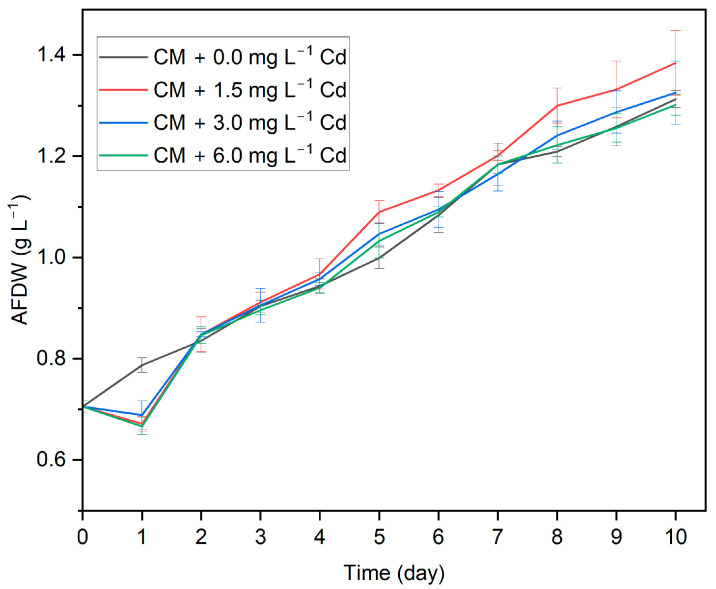
Growth profile of *G. sulphuraria* in the medium with different concentrations of Cd. Data points represent the average ± SD of *n* = 3 biological replicates.

Significant morphological and metabolic changes are known to occur in algal cells upon exposure to HMs, which in turn affect algal growth. The effects on the growth of a particular algal strain depend on the specific metal and its concentration. To investigate the impact of Cd ions on the growth of *G. sulphuraria*, this algal strain was grown in CM with varying concentrations of Cd. The growth curve for *G. sulphuraria* in the different media compositions is shown in Figure 5. The growth curve of *G. sulphuraria* in the medium containing Cd exhibits a lag, exponential, and stationary growth phase.

On the other hand, with the absence of Cd in the medium, the growth curve had no lag phase. In all the tested concentrations, there is no significant difference (*p* > 0.05) (F = 4.68, *p* = 0.096 (1.5 mg L^−1^)), (F = 1.4, *p* = 0.72 (3 mg L^−1^)), (F = 4.26, *p* = 0.106 (6 mg L^−1^)) in growth on day 10 compared to the control group; although there has been some variation of AFDW in the growth curve. Cd inhibited the growth of *G. sulphuraria* in a concentration-dependent manner. In other words, when the concentration of Cd in the medium increases from 1.5 mg L^−1^ to 6 mg L^−1^ Cd, the algal growth rate decreases, respectively. *G. sulphuraria* retains a higher growth rate (*p* ≤ 0.05) (F = 0.779, *p* = 0847) when exposed to an initial Cd concentration of 1.5 mg L^−1^ compared to 6 mg L^−1^.

## 4. Discussion

This study sought to determine whether *G. sulphuraria* could effectively remove Cd throughout a ten-day incubation period. In addition, this study seeks to quantify the total amount of Cd removed, separating it into intracellular and extracellular components. The results demonstrated that *G. sulphuraria* effectively removed Cd from the various medium compositions.

Results from our study showed that the removal efficiency of Cd is 29.52%, 30.00%, and 18.88% in the medium added with 1.5, 3, and 6 mg L^−1^ Cd, respectively; with corresponding sorption capacities of 0.63, 1.3, and 1.59 mg g^−1^ of dry biomass. These results are comparable to the results obtained in other literature using different algal strains that survive in less acidic pH. Most studies on Cd bioremoval have used green algal strains. According to the research conducted by Chandrashekharaiah et al. [9], microalgae *C. pyrenoidosa* and *S. acutus* were used to remove 1.5 mg L^−1^ Cd from the solution and achieved the results with 45.45% and 57.14% removal efficiency and 0.27 and 0.25 mg g^−1^ sorption capacity, respectively. Duque et al. [34] used two algal strains—*Chlorella* sp. and *Scenedesmus* sp.—to remove a Cd concentration of 1 mg L^−1^, and the corresponding removal efficiencies were 8.07% and 5.13%. When the concentration increased to 7 mg L^−1^, the removal efficiency increased to 8.60% and 32.74%, respectively, for *Chlorella* sp. and *Scenedesmus* sp. Abinandan et al. [35] used acid-tolerant microalgae *Heterochlorella* sp. MAS3 and *Desmodesmus* sp. MAS1 to remove 1 and 2 mg L^−1^ of Cd at an acidic pH of 3.5 and achieved the results of 0.16 to 0.36 mg g^−1^ and 0.37 to 0.77 mg g^−1^ sorption capacity, respectively. According to our research, the removal efficiency of Cd is identical at 1.5 and 3 mg L^−1^ initial Cd concentration. However, the removal efficiency declines as the concentration increases from 3 mg L^−1^ to 6 mg L^−1^. This may be because as the concentration of Cd increases in the solution, its toxicity also increases; reducing the overall health and viability of the algal cells and limiting their efficiency in removing Cd. Similar results were obtained from other researchers. Using the green algae *Cladophora fractureta* and varying Cd concentrations of 0.1 mg L^−1^ to 1 mg L^−1^, Ji et al. [36] investigated the Cd removal effectiveness. Maximum removal effectiveness of 97% occurred at lower Cd concentrations, while the sorption capacity ranged between 0.005 to 0.24 mg g^−1^ with increases at higher Cd concentrations. In a study conducted by Shamshad et al. [37], the efficacy of removing Cd from green macroalgae *Oedogonium westti* varied from 55% to 95% across initial Cd concentrations of 0.5 to 2.0 mg L^−1^, with the greatest removal observed at lower concentrations.

There has been very little research conducted into the potential of red algal strains for Cd bioremoval. With the use of the *G. sulphuraria* IPPAS P-513 strain, Ostroumov et al. [38] found a reduction of the average Cd level by 24% after 30 days of incubation. Another investigation was conducted by Isachsen et al. [39], utilizing two distinct strains of the red algal strain *Cyanidioschyzon merolae*; namely, MS1 and 10D. After seven days of treatment for the Cd removal, strain 10D removed 31.55% of Cd, and strain MS1 only removed 1.16% of Cd. Removal efficiency not only depends on the type of HM but also on the specific algal species. Studies were conducted by Folgar et al. [40] using *Dunaliella salina,* and Pérez-Rama et al. [5] using *Tetraselmis suecica* for the bioremoval of Cd in the same experimental conditions. The results indicate that the removal efficiency of Cd ranges from 2.9% to 11.3% using *Dunaliella salina* and 7.7% to 98.4% while using *Tetraselmis suecica.* The comparisons between the findings of this research and those reported in the various literature regarding the removal of Cd from aqueous solutions using different green and red algal strains under varying initial Cd concentrations and pH conditions are presented in Table 1.

This study also explores whether total Cd removal occurs via extracellular or intracellular mechanisms. The process of HM uptake by living microalgal cells consists of extracellular and intracellular processes. During the extracellular process, biosorption occurs when metal ions are absorbed onto cell surfaces. This process is relatively fast and may or may not entail metabolic processes. During the intracellular process, bioaccumulation occurs where metal ions enter live cells and eventually build up in the cytoplasm. This relatively slow process requires metabolic activity [16,45,46,47]. 

In our study, the extracellular removal percentage in the medium with initial Cd concentrations of 1.5 mg L^−1^, 3 mg L^−1^, and 6 mg L^−1^ were 82.31%, 82.27%, and 81.31%, respectively; indicating that the majority of Cd removal occurred via an extracellular pathway. Few studies have found similar results. Chandrashekharaiah et al. [9] used two microalgae—*C. pyrenoidosa* and *S. acutus*—to remove 1.5 mg L^−1^ Cd from the solution; and the results indicated that *C. pyrenoidosa* accounted for 3% intracellular and 97% extracellular of the total Cd removed. Similarly, *S. acutus* exhibited intracellular Cd removal of 1.5% and extracellular removal of 98.5%. While studying Cd-tolerant microalgal strains in Nordic habitats, Plohn et al. [45] found that *Chlorella vulgaris* and *Coelastrella* sp. effectively removed the Cd. Analysis of biomass using Fourier Transform Infrared Spectroscopy revealed that the exterior cell wall was the primary site of Cd removal and the carboxylic moieties found in cell walls were identified as responsible for Cd removal. In some research, most Cd removal occurs via intracellular accumulation as opposed to our research. Research conducted by Torres et al. [48] in *Phaeodactylum tricornutum* cultures and Pérez-Rama et al. [5] in *Tetraselmis suecica* indicates that the amount of Cd that these microalgae take in through passive adsorption into their cell surfaces is significantly lower than the amount of Cd that is removed from within their cells. *Chlorella minutissima* UTEX2341 showed remarkable Cd removal efficacy in heterotrophic conditions, achieving an efficiency of 74.34%. According to the transmission electron microscopy results, the main mechanism for Cd elimination is intracellular accumulation, with the addition of extracellular immobilization [49]. 

This study also examines the influence of Cd ions on the growth of *G. sulphuraria* and the subsequent production of algal biomass. Our investigation found that *G. sulphuraria* growth was initially inhibited and then started to increase suggesting that *G. sulphuraria* requires an initial acclimation period to adapt to HM contamination. Initially, Cd temporarily inhibits growth due to stress responses and cellular damage. Over time, the algae activate detoxification mechanisms, repair cellular damage, and lead to improved growth; adjusting their physiology to better cope with the Cd stress, which can result in higher growth at later stages. Other researchers have found similar results. According to Bajguz et al. [50], the growth and chemical composition of *Chlorella vulgaris*—including chlorophyll, monosaccharides, and protein content—were shown to be reduced during the first 48 h of cultivation when Cd was added. In this research, the algal growth rate is higher at lower Cd concentrations. As the Cd level increases, more significant cellular damage occurs which requires more energy and resources for detoxification and cell repair, resulting in reduced growth. This finding resonates with the results obtained by Torres et al. [3], which showed that there are significant effects on the growth of *Phaeodactylum tricornutum* while exposed to Cd concentrations above 5 mg L^−1^. Similar results were obtained by Chandrashekharaiah et al. [9] in which the growth of *Chlorella pyrenoidosa* and *Scenedesmus acutus* was comparable to the control medium while exposed to 1.5 mg L^−1^ of Cd, and inhibition occurred when exposed beyond 5 mg L^−1^. Furthermore, when the concentration of Cd in the medium increases, the algal growth rate decreases. Similar results were obtained by Pérez-Rama et al. [5], where growth inhibition of *Tetraselmis suecica* was proportional to a concentration between 0.6 mg L^−1^ to 45 mg L^−1^, with a lower growth rate at higher Cd concentration. 

There may be various reasons why HMs cause toxic effects on the algal cells. According to Pinto et al. [51], the primary cause by which HMs induce toxicity is through their oxidative effect on intracellular components, which leads to damage of the chloroplast and negatively affects the photosynthesis process. Antioxidants are very effective for protecting algal cells from oxidative stress caused by HMs and other pollutants, but their effectiveness decreases when there is an increase in HM concentration within the cells. Toxic effects on the microalgal cell can also arise when HMs block the functional groups of enzymes and the transport routes for essential nutrients [52]. Multiple intracellular and extracellular detoxification pathways mitigate HM toxicity within the microalgal cell. These pathways involve biosorption on the cell wall, biosorption on the extracellular polymeric substances, bioaccumulation within intracellular compartments, and the transformation of HMs by algal cells [16]. Very few studies were performed regarding toxic effects of Cd ions on algal cells. According to Vymzal et al. [53], due to the strong attraction of Cd to -SH groups found in proteins and enzymes, it inhibits a variety of cellular activities; including growth, photosynthesis, respiration rate, and others. Cd reacts with oxygen molecules and produces reactivated oxygen species (ROS). The harmful effects of ROS include lipid membrane peroxidation, polysaccharide depolymerization, protein denaturation, and DNA damage [40]. 

Although *G. Sulphuraria* can be used to remediate HMs, disposal of microalgal biomass after treatment is a major challenge when it comes to large-scale operations. Microalgal biomass, which is rich in HM, has always been a major problem when it comes to safe disposal. Due to leaching and temperature fluctuations, HMs that are present in biomass may be released into the adjacent environment and may cause secondary pollution. Various methods have been used in the past to manage HM-contaminated biomass. Bioleaching is an environmentally friendly and cost-effective method for extracting metals from various mineral and waste sources. Metal-free biomass can be converted into value-added products using various processes such as thermochemical processing [54] and hydrothermal liquefaction [55]. Furthermore, some research has successfully converted HM-contaminated biomass into energy products such as bio-oil, charcoal, and biogas using thermochemical treatment methods. Converting contaminated biomass into value-added commodities reduces carbon emissions and secondary pollution. Enzymes, bioenergy, biopolymers, pigments, sugars, etc., are all examples of novel products that have potential societal applications [56]. There is strong economic feasibility in producing organic acids from the HM polluted biomass [57]. Converting HM-contaminated biomass into metal-loaded biochar compounds is another approach to biomass management and pyrolysis is a promising method for this conversion [58]. Many useful compounds derived from biochar have found applications in energy storage, catalysis, adsorption, and other fields.

## 5. Conclusions

This study provides valuable insights for the removal of low concentrations of Cd (II) (0 mg L^−1^ to 6 mg L^−1^) under acidic pH conditions using acidophilic microalga *G. sulphuraria* under controlled laboratory conditions. Time course Cd removal, total Cd removal, extracellular Cd removal, and intracellular Cd removal were calculated during the ten-day incubation period. Results obtained from the study showed that *G. sulphuraria* can grow in the Cd-containing medium with initial growth inhibition. For the three Cd concentrations given, *G. sulphuraria* removed the Cd in the range of 18.88% to 30.00% with a sorption capacity of 0.63 mg g^−1^ to 1.59 mg g^−1^ dry biomass. Cd removal by *G. sulphuraria* is largely due to a non-metabolic process since the amount of Cd eliminated intracellularly was significantly lower than the amount adsorbed on the cell surface. While this study contributes valuable insights for the removal of Cd from aqueous solution using *G. sulphuraria*, there are still unexplored areas that require further investigation. These include understanding the detailed mechanisms behind the removal of Cd, evaluating the feasibility of scaling up this process for large-scale applications, assessing the system’s performance with real wastewater effluents, and investigating the effects of different parameters—such as the presence of organic compounds and changes in pH, algal biomass, and temperature—in removal efficiency.

## Figures and Tables

**Table 1:** Removal efficiency of Cd using live algae from this study vs. literature review.

Algal Strain	Initial Cd Concentration (mg L^−1^)	pH	Sorption Capacity (mg g^−1^)	Removal Efficiency (%)	References
Green Algae					
*Scenedesmus acutus*	1.5	7.0	0.25	57.14	[9]
*Chlorella pyrenoidosa*	1.5	7.0	0.27	45.45	[9]
*Chlorella* sp.	1.0–7.0			8.07–8.60	[34]
*Scenedesmus* sp.	1.0–7.0			5.13–32.74	[34]
*Oedogonium westii*	0.50–2.00	5.0	0.974	55.00–95.00	[37]
*Ulva lactuca*	0.01–0.2		0.347	57.00–96.00	[41]
*Ulva lactuca*	0.20	7.8	0.018	56.00	[42]
*Desmodesmus* sp. MAS1	1.00–2.00	3.5	0.37–0.77		[35]
*Heterochlorella* sp. MAS3	1.00–2.00	3.5	0.16–0.36		[35]
*Didymogenes palatina* XR	2.0	6.0	7.41	87.99	[43]
*Cladophora fracta*	0.1–1.0	5.0	0.05–0.24	97.00	[36]
*Dunaliella salina*	5.0–120.0	7.8		2.9–11.3	[40]
*Tetraselmis suecica*	0.6–45.0	7.8		7.7–98.4	[5]
Red Algae					
*Cyanidioschyzon merolae* 10D	1.00	1.75	-	31.55	[39]
*Cyanidioschyzon merolae* MS1	5.00	1.75	-	1.16	[39]
*Galdieria sulphuraria* *IPPAS P-513*	5.00	2.7	-	24.00	[38]
*Galdieria sulphuraria* *CCMEE 5587.1*	1.25–5.00	2.5	0.64–1.45	19.09–49.80	[44]
*Galdieria sulphuraria* *CCMEE 5587.1*	1.5–6.00	2.5	0.63–1.59	18.89–30.00	This study

## Data Availability

The data presented in this study are available on request from the corresponding author. The data are not publicly available due to the continuation of a follow-up study by the authors.

## References

[1] Abdi O., Kazemi M. (2015). A review study of biosorption of heavy metals and comparison between different biosorbents. J. Mater. Environ. Sci..

[2] Mehta S., Gaur J. (2005). Use of algae for removing heavy metal ions from wastewater: Progress and prospects. Crit. Rev. Biotechnol..

[3] Torres E., Cid A., Herrero C., Abalde J. (1998). Removal of cadmium ions by the marine diatom *Phaeodactylum tricornutum* Bohlin accumulation and long-term kinetics of uptake. Bioresour. Technol..

[4] Tukaj Z., Baścik-Remisiewicz A., Skowroński T., Tukaj C. (2007). Cadmium effect on the growth, photosynthesis, ultrastructure and phytochelatin content of green microalga *Scenedesmus armatus*: A study at low and elevated CO_2_ concentration. Environ. Exp. Bot..

[5] Pérez-Rama M., Alonso J.A., López C.H., Vaamonde E.T. (2002). Cadmium removal by living cells of the marine microalga *Tetraselmis suecica*. Bioresour. Technol..

[6] Martin S., Griswold W. (2009). Human health effects of heavy metals. Environ. Sci. Technol. Briefs Citiz..

[7] Kinuthia G.K., Ngure V., Beti D., Lugalia R., Wangila A., Kamau L. (2020). Levels of heavy metals in wastewater and soil samples from open drainage channels in Nairobi, Kenya: Community health implication. Sci. Rep..

[8] USEPA Effluent Guidelines Database. https://www.epa.gov/eg/effluent-guidelines-database.

[9] Chandrashekharaiah P., Sanyal D., Dasgupta S., Banik A. (2021). Cadmium biosorption and biomass production by two freshwater microalgae *Scenedesmus acutus* and *Chlorella pyrenoidosa*: An integrated approach. Chemosphere.

[10] Gupta V., Rastogi A. (2008). Equilibrium and kinetic modelling of cadmium (II) biosorption by nonliving algal biomass *Oedogonium* sp. from aqueous phase. J. Hazard. Mater..

[11] Zeraatkar A.K., Ahmadzadeh H., Talebi A.F., Moheimani N.R., McHenry M.P. (2016). Potential use of algae for heavy metal bioremediation, a critical review. J. Environ. Manag..

[12] Karki B.K., Baniya S., Kharel H.L., Angove M.J., Paudel S.R. (2024). Urban wastewater management in Nepal: Generation, treatment, engineering, and policy perspectives. H_2_Open J..

[13] Kumar K.S., Dahms H.-U., Won E.-J., Lee J.-S., Shin K.-H. (2015). Microalgae—A promising tool for heavy metal remediation. Ecotoxicol. Environ. Saf..

[14] Wang J., Chen C. (2009). Biosorbents for heavy metals removal and their future. Biotechnol. Adv..

[15] Jacinto M.L.J., David C.P.C., Perez T.R., De Jesus B.R. (2009). Comparative efficiency of algal biofilters in the removal of chromium and copper from wastewater. Ecol. Eng..

[16] Danouche M., El Ghachtouli N., El Arroussi H. (2021). Phycoremediation mechanisms of heavy metals using living green microalgae: Physicochemical and molecular approaches for enhancing selectivity and removal capacity. Heliyon.

[17] Nelson N., Huynh T., Nguyen M., Choudhury N., Dao D., Bougere J., Crouere J., Baroun A. Heavy metal removal via phycoremediation. Proceedings of the 2020 Waste-Management Education Research (WERC).

[18] Leong Y.K., Chang J.-S. (2020). Bioremediation of heavy metals using microalgae: Recent advances and mechanisms. Bioresour. Technol..

[19] Selvaratnam T., Kharel H.L., Tan M. Algal-Based Bioremediation of Landfill Leachate. Proceedings of the 2022 AIChE Annual Meeting.

[20] Sirakov M., Palmieri M., Iovinella M., Davis S.J., Petriccione M., di Cicco M.R., De Stefano M., Ciniglia C. (2021). Cyanidiophyceae (Rhodophyta) Tolerance to Precious Metals: Metabolic Response to Palladium and Gold. Plants.

[21] Iovinella M., Lombardo F., Ciniglia C., Palmieri M., Di Cicco M.R., Trifuoggi M., Race M., Manfredi C., Lubritto C., Fabbricino M. (2022). Bioremoval of Yttrium (III), Cerium (III), Europium (III), and Terbium (III) from Single and Quaternary Aqueous Solutions Using the Extremophile Galdieria sulphuraria (Galdieriaceae, Rhodophyta). Plants.

[22] Cho Y.-L., Lee Y.-C., Hsu L.-C., Wang C.-C., Chen P.-C., Liu S.-L., Teah H.-Y., Liu Y.-T., Tzou Y.-M. (2020). Molecular mechanisms for Pb removal by Cyanidiales: A potential biomaterial applied in thermo-acidic conditions. Chem. Eng. J..

[23] Minoda A., Sawada H., Suzuki S., Miyashita S.-i., Inagaki K., Yamamoto T., Tsuzuki M. (2015). Recovery of rare earth elements from the sulfothermophilic red alga *Galdieria sulphuraria* using aqueous acid. Appl. Microbiol. Biotechnol..

[24] Čížková M., Mezricky P., Mezricky D., Rucki M., Zachleder V., Vítová M. (2021). Bioaccumulation of rare earth elements from waste luminophores in the red algae, *Galdieria phlegrea*. Waste Biomass Valorization.

[25] Oesterhelt C., Schmälzlin E., Schmitt J.M., Lokstein H. (2007). Regulation of photosynthesis in the unicellular acidophilic red alga *Galdieria sulphuraria*. Plant J..

[26] Kharel H.L., Shrestha I., Tan M., Nikookar M., Saraei N., Selvaratnam T. (2023). Cyanidiales-Based Bioremediation of Heavy Metals. BioTech.

[27] Selvaratnam T., Pegallapati A., Montelya F., Rodriguez G., Nirmalakhandan N., Lammers P.J., Van Voorhies W. (2015). Feasibility of algal systems for sustainable wastewater treatment. Renew. Energy.

[28] Pan S., Dixon K.L., Nawaz T., Rahman A., Selvaratnam T. (2021). Evaluation of *Galdieria sulphuraria* for nitrogen removal and biomass production from raw landfill leachate. Algal Res..

[29] Rahman A., Pan S., Houston C., Selvaratnam T. (2021). Evaluation of *Galdieria sulphuraria* and *Chlorella vulgaris* for the Bioremediation of Produced Water. Water.

[30] Kumar M., Kushwaha A., Goswami L., Singh A.K., Sikandar M. (2021). A review on advances and mechanism for the phycoremediation of cadmium contaminated wastewater. Clean. Eng. Technol..

[31] Cui M., Jang M., Cho S.-H., Khim J., Cannon F.S. (2012). A continuous pilot-scale system using coal-mine drainage sludge to treat acid mine drainage contaminated with high concentrations of Pb, Zn, and other heavy metals. J. Hazard. Mater..

[32] Palma H., Killoran E., Sheehan M., Berner F., Heimann K. (2017). Assessment of microalga biofilms for simultaneous remediation and biofuel generation in mine tailings water. Bioresour. Technol..

[33] Toplin J.A., Norris T.B., Lehr C.R., McDermott T.R., Castenholz R.W. (2008). Biogeographic and phylogenetic diversity of thermoacidophilic cyanidiales in Yellowstone National Park, Japan, and New Zealand. Appl. Environ. Microbiol..

[34] Duque D., Montoya C., Botero L.R. (2019). Cadmium (Cd) tolerance evaluation of three strains of microalgae of the genus Ankistrodesmus, C hlorella and Scenedesmus. Rev. Fac. Ing. Univ. Antioq..

[35] Abinandan S., Subashchandrabose S.R., Venkateswarlu K., Perera I.A., Megharaj M. (2019). Acid-tolerant microalgae can withstand higher concentrations of invasive cadmium and produce sustainable biomass and biodiesel at pH 3.5. Bioresour. Technol..

[36] Ji L., Xie S., Feng J., Li Y., Chen L. (2012). Heavy metal uptake capacities by the common freshwater green alga *Cladophora fracta*. J. Appl. Phycol..

[37] Shamshad I., Khan S., Waqas M., Asma M., Nawab J., Gul N., Raiz A., Li G. (2016). Heavy metal uptake capacity of fresh water algae (*Oedogonium westti*) from aqueous solution: A mesocosm research. Int. J. Phytoremediat..

[38] Ostroumov S., Tropin I., Kiryushin A. (2018). Removal of cadmium and other toxic metals from water: Thermophiles and new biotechnologies. Russ. J. Gen. Chem..

[39] Isachsen I. (2022). Cadmium Tolerance in the Thermo-Acidophilic Red Alga C. merolae, Possible Mechanisms and Implications for Bioremediation.

[40] Folgar S., Torres E., Pérez-Rama M., Cid A., Herrero C., Abalde J. (2009). Dunaliella salina as marine microalga highly tolerant to but a poor remover of cadmium. J. Hazard. Mater..

[41] Henriques B., Rocha L.S., Lopes C.B., Figueira P., Duarte A., Vale C., Pardal M., Pereira E. (2017). A macroalgae-based biotechnology for water remediation: Simultaneous removal of Cd, Pb and Hg by living Ulva lactuca. J. Environ. Manag..

[42] Henriques B., Teixeira A., Figueira P., Reis A.T., Almeida J., Vale C., Pereira E. (2019). Simultaneous removal of trace elements from contaminated waters by living *Ulva lactuca*. Sci. Total Environ..

[43] Wang Z., Xia L., Song S., Farías M.E., Li Y., Tang C. (2021). Cadmium removal from diluted wastewater by using high-phosphorus-culture modified microalgae. Chem. Phys. Lett..

[44] Kharel H.L., Shrestha I., Tan M., Selvaratnam T. (2023). Removal of cadmium and lead from synthetic wastewater using *Galdieria sulphuraria*. Environments.

[45] Plöhn M., Escudero-Oñate C., Funk C. (2021). Biosorption of Cd(II) by Nordic microalgae: Tolerance, kinetics and equilibrium studies. Algal Res..

[46] Priyadarshini E., Priyadarshini S.S., Pradhan N. (2019). Heavy metal resistance in algae and its application for metal nanoparticle synthesis. Appl. Microbiol. Biotechnol..

[47] Flouty R., Estephane G. (2012). Bioaccumulation and biosorption of copper and lead by a unicellular algae *Chlamydomonas reinhardtii* in single and binary metal systems: A comparative study. J. Environ. Manag..

[48] Torres E., Cid A., Herrero C., Abalde J. (2000). Effect of cadmium on growth, ATP content, carbon fixation and ultrastructure in the marine diatom *Phaeodactylum tricornutum* Bohlin. Water Air Soil Pollut..

[49] Yang J., Cao J., Xing G., Yuan H. (2015). Lipid production combined with biosorption and bioaccumulation of cadmium, copper, manganese and zinc by oleaginous microalgae *Chlorella minutissima* UTEX2341. Bioresour. Technol..

[50] Bajguz A. (2011). Suppression of Chlorella vulgaris growth by cadmium, lead, and copper stress and its restoration by endogenous brassinolide. Arch. Environ. Contam. Toxicol..

[51] Pinto E., Sigaud-kutner T.C., Leitao M.A., Okamoto O.K., Morse D., Colepicolo P. (2003). Heavy metal–induced oxidative stress in algae 1. J. Phycol..

[52] Napan K., Teng L., Quinn J.C., Wood B.D. (2015). Impact of heavy metals from flue gas integration with microalgae production. Algal Res..

[53] Vymazal J. (1987). Toxicity and accumulation of cadmium with respect to algae and cyanobacteria: A review. Toxic. Assess..

[54] Mulbry W., Westhead E.K., Pizarro C., Sikora L. (2005). Recycling of manure nutrients: Use of algal biomass from dairy manure treatment as a slow release fertilizer. Bioresour. Technol..

[55] Selvaratnam T., Pegallapati A., Reddy H., Kanapathipillai N., Nirmalakhandan N., Deng S., Lammers P. (2015). Algal biofuels from urban wastewaters: Maximizing biomass yield using nutrients recycled from hydrothermal processing of biomass. Bioresour. Technol..

[56] Chugh M., Kumar L., Shah M.P., Bharadvaja N. (2022). Algal Bioremediation of heavy metals: An insight into removal mechanisms, recovery of by-products, challenges, and future opportunities. Energy Nexus.

[57] Guan C.-Y., Chen S.S., Lee T.-H., Yu C.-P., Tsang D.C. (2020). Valorization of biomass from plant microbial fuel cells into levulinic acid by using liquid/solid acids and green solvents. J. Clean. Prod..

[58] Chai Y., Chen A., Bai M., Peng L., Shao J., Yuan J., Shang C., Zhang J., Huang H., Peng C. (2022). Valorization of heavy metal contaminated biomass: Recycling and expanding to functional materials. J. Clean. Prod..

